# Global trends and research hotspots of PCSK9 and cardiovascular disease: a bibliometric and visual analysis

**DOI:** 10.3389/fcvm.2024.1336264

**Published:** 2024-06-03

**Authors:** Masong Tang, Sen Yang, Junying Zou, Meng Li, Yan Sun, Mengqi Wang, Wanhan Li, Junhui He, Ying Chen, Zhanyou Tang

**Affiliations:** ^1^ Department of Basic Medical Sciences, Medical School, University of South China, Hengyang, Hunan, China; ^2^ Department of Urology, Hunan University of Medicine General Hospital, Huaihua, Hunan, China; ^3^ Department of Gynecologic, Hunan University of Medicine General Hospital, Huaihua, Hunan, China

**Keywords:** cardiovascular disease, PCSK9, WOS, bibliometrics, visual analysis

## Abstract

**Background:**

Cardiovascular disease (CVD) is a prevalent non-communicable disease globally and holds the position of being the primary cause of mortality worldwide. Consequently, considerable focus has been directed towards the prevention and management of CVD. PCSK9, a frequently targeted element in the treatment and prevention of CVD, can reduce cardiovascular risk by effectively lowering lipid levels even in the context of statin therapy. It also exhibits substantial potential in the diagnosis and treatment of familial hypercholesterolemia from genetic aspects. This bibliometric study aims to analyze and visualize the global trends and emerging hotspots of PCSK9 and CVD researches and provide researchers with new perspectives in further studies.

**Methods:**

The data was obtained from the Web of Science Core Collection database. A total of 2,474 publications related to PCSK9 and CVD published between January 2006 and July 2023 were included. The VOSviewer was used to analyze most-cited references, co-authorship, co-citation, co-occurrence and generate a collaborative network map of authors, countries, and institutions. CiteSpace was used to analyze author and institution centroids, keyword bursts, and timeline graphs.

**Result:**

A total of 2,474 articles related to CVD and PCSK9 were included. The number of articles and citations show an increasing trend from year to year. Publications were mainly from the United States. The most active institution was Amgen Inc. Watts, Gerald F. was the most prolific author. Atherosclerosis was the most published journal. Literature co-citation and keyword co-occurrence revealed that early studies focused on the lipid-lowering effects of PCSK9 inhibitors in the context of statins therapy, long-term efficacy, adverse effects, LDLR, diagnosis and treatment of familial hypercholesterolemia. In recent years, myocardial ischemic protection, CRISPR-based editing, and new therapeutic strategies for arteriosclerotic cardiovascular disease have gotten wide attention. The protein convertase, inflammation, beta-polyacetate, and inclisiran may be the important future research directions.

**Conclusion:**

This study analyses the current status and global trends in the CVD and PCSK9 studies comprehensively, which may provide researchers and policymakers with new and comprehensive perspectives on in this field of research.

## Introduction

Cardiovascular diseases (CVDs) are the most prevalent non-communicable diseases worldwide (1) including ischemic heart disease, stroke, heart failure, peripheral artery disease, and several other heart and vascular diseases. CVDs are the leading causes of death and a major cause of reduced life quality worldwide. In 2017, CVDs caused approximately 17.8 million deaths globally, which are equivalent to 330 million years of life lost and 35.6 million years of life lived with disability, more than three-quarters of which occurred in low- and middle-income countries (2, 3).

Dyslipidemia is defined as abnormal levels of lipids and lipoproteins in the blood, with significant elevation of low-density lipoprotein cholesterol (LDL-C) being a key manifestation. Globally, elevated LDL has been proved as an independent risk predictor for many cardiovascular and cerebrovascular events. Therefore, keeping normal lipid levels have important prognostic implications for reducing the burden of stroke and myocardial infarction (MI) (4, 5). A systematic review and meta-analysis estimated that each 10% reduction in LDL-C reduced the risk of all strokes by 15.6% and carotid Intima-Media Thickness (IMT) by 0.73% per year (6). Meanwhile, LDL and apolipoprotein B (apo B) play a central role in the initiation and progression of atherosclerosis (7). In fact, the risk of suffering a heart attack seemingly appers to double for each decade of exposure to the same plasma LDL levels once surpassing the cumulative LDL exposure threshold. This highlights the significance of dyslipidemia in cardiovascular diseases development.

According to the JACC Health Promotion Series recommendations, a lifelong strategy of maintaining lower lipid levels and minimizing cumulative exposure to LDL and apo B may significantly delay the course of atherosclerotic plaque progression and reduce the lifetime risk of cardiovascular events substantially. Many researchers have certified that lowering the levels of LDL and apo B can reduce the risk of cardiovascular events by about 20%/mmol/L (8–11).

At present, common lipid-lowering treatments comprise statins, PCSK9 inhibitors, polydatin, resveratrol, and certain nutraceuticals (12–16). Researches indicate that statins reduced total cholesterol by 20% and LDL by 26% (12). There is increasing evidence that statins play a crucial role in decreasing the likelihood of cardiovascular events. While Resveratrol, a natural polyphenol found in red wine, is utilized to control blood lipid levels in order to lower the risk of hyperlipidemia and atherosclerosis (17). As a glucoside derivative of resveratrol, polydatin are also effective in lowering serum total cholesterol (TC), low-density lipoprotein cholesterol (LDL-C), and triglycerides (TG) (18–20). PCSK9 is a key factor in LDL-C homeostasis, and its related targets have been utilized in lipid-lowering strategies (13). Nutraceuticals like berberine, curcumin, indigo, and ω-3 fatty acids exhibit antioxidant and hypolipidemic effects, possibly by reducing PCSK9 expression (21, 22).

In these researches, PCSK9-related targets have been received extensive attention. In 2003, the proprotein convertase subtilisin/kexin type 9 was first identified in the brain as the ninth member of the convertase family and standardized under the name PCSK9 (23). Research has demonstrated the vital involvement of PCSK9 in the LDL receptor's life cycle (13). Early studies focused on its role in the liver in regulating LDL metabolism. PCSK9 is secreted by hepatocytes and binds to the epidermal growth factor-like repeat sequence A (EGF-A) structural domain of the LDL receptor. The presence of PCSK9 prevents conformational changes in the LDL receptor when the PCSK9-LDL receptor complex is internalized. Subsequently, the complex moves with LDL to the lysosome where it is destroyed. This regulates the circulation of low-density lipoprotein cholesterol (LDL-C) on the surface of hepatocytes and controls its concentration (13). The first clinical evidence linking PCSK9 and cardiovascular diseases (CVD) discovered: patients with gain-of-function mutations in PCSK9 showed significantly increased serum levels of LDL-C, decreased levels of LDL receptors on hepatocytes, and was prone to experiencing premature cardiovascular events (24). Conversely, patients with loss-of-function mutations exhibited low serum PCSK9 levels and a remarkable reduction of more than 40% in LDL cholesterol (24). This evidence underscores the pivotal role of PCSK9 in regulating LDL cholesterol levels and its implications for cardiovascular health. In the fields of genetics and mutations, reducing PCSK9 levels helps lower blood lipid levels to normal, significantly reducing cardiovascular risk (25–28).

The first PCSK9 inhibitor was invented in 2015, and it was rapidly adopted in the clinic for its potent lipid-lowering effect and safety profile (29). At present, alirocumab, evolocumab, and inclisiran are the primary PCSK9 inhibitors used in clinical practice. They are primarily employed for treating clinical hyperlipidemia, atherosclerosis (AS), and related ischemic cardiovascular diseases. They are also alternatives to statins and ezetimibe (25–28). Furthermore, a meta-analysis showed that the PSCK9 inhibitors could reduce 19% the incidence of myocardial infarctions and 25% the incidence of stroke (30). Study discovered that elevated plasma levels of PCSK9 diminished the efficacy of statins during statin use (31). PCSK9 antibodies have been identified as emerging agents that can reduce the risk of cardiovascular diseases, either as supplements to statins or as an alternative when statins are ineffective. According to the current guidelines of the American College of Cardiology and the American Heart Association in the Cardiovascular Regression Study, non-statin cholesterol-lowering therapies, such as PCSK9i, may be added to patients at very high risk of major adverse cardiovascular events (MACE) when LDL-C levels remain ≥70 mg/dl (32).

However, the PCSK9 gene is also expressed in multiple organs, including liver, kidney, small intestine, and cancer cells (31). In recent years, extrahepatic studies on PCSK9 have become a new direction of interest involving cardiomyocyte, macrophage, endothelial cell, and cancer cell metabolism, thus revealing the existence of a wider range of applications for PCSK9 targets. Immune checkpoint inhibitors (ICIs), as an emerging strategy for tumor therapy (33, 34), are associated with ICIs-induced cardiovascular disease-related side effects. These side effects are rare but clinically significant, include concurrent fatal myocarditis, vasculitis, arrhythmias, fibrosis, and heart failure (35, 36). ICIs treatment induces in myocardial tissues the production of damage associated molecular patterns (DAMPs), expression of NLRP3 and MyD88. NLRP3 drives cytoplasmic damage, hypertrophy, and inflammation through overproduction of cytokines and hs-CRP (31). PCSK9i-based drugs may be a key cardioprotective strategy for arteriosclerotic cardiovascular diseases (ASCVD) in cancer patients treated with ICIs by acting on pathways and cytokine-mediated effects such as NLRP-3/IL-1 overexpression, My-D88/TLR4 (37, 38). ICIs-treated patients are more likely to be exposed to the risk of atherosclerosis (39). PCSK9 plays a key role in platelet aggregation and adhesion, endothelial dysfunction and atherosclerosis. PCSK9-associated targets may have a role in reducing this risk (39). Studies reveal that PCSK9i may exert effects in cancer patients with ICIs treatment strategy by enhancing the efficacy of immunotherapy, inhibiting apoptosis resistance mechanisms, reducing the risk of stenotic plaque destabilization, and decreasing the risk of atherosclerosis induced by ICIs (31). Therefore, it is conceivable that cancer patients treated with ICIs, particularly those diagnosed with ASCVD, could potentially benefit from PCSK9 therapy.

PCSK9 has been widely studied and applied in the detection screening, prevention, and treatment of cardiovascular diseases. However, it still lacks precise information on countries, authors, publications, institutions, journals, and keywords related to cardiovascular diseases and researches in the field of PCSK9.

Bibliometrics is a research method that employs mathematical and statistical techniques to thoroughly analyze specific documents (40). Bibliometric analysis enables a comprehensive evaluation of the quality and quantity of the scientific results. With the increasing popularity of its methods and tools, it is now possible to collect and analyze appropriate literature to effectively judge the development status of the discipline and predict its development prospects (40). These studies can characterize publication trends, including impact and citations, and also reflect health policy decisions, healthcare resource inputs, and social phenomena (41). To assess the impact of CVD and PCSK9 researches on global scientific outputs, as well as to make contributions to the prevention and control of CVD, we conducted a bibliometric analysis using the Web of Science database indexes. We revealed the main contributors and the current status of the researches and discovered the trends and prospects in this field.

## Materials and methods

### Methodology

Bibliometrics is a method of analyzing publications from quantitative and qualitative perspectives to determine their output and status. It was formed as a separate discipline in 1969 (42) and was widely used in literature analysis (43). Devey summarized that “bibliometrics is to scientific papers what epidemiology is to patients” (44). It can help researchers quickly understand the trajectory of a topic by tracking its spread through the literature. It can also characterize actively and heavily downloaded journal articles to assess their impacts (45).

Through the analysis process, we can obtain detailed information concerning authors, keywords, journals, countries, institutions, and references in a specific field, enabling the unveiling of the field's development through bibliometric analysis (46). The visualization can provide an intuitive presentation and a good complement to the results of bibliometric analysis. Furthermore, visualization enables the exploration of connections among information, such as similarities in research topics among different authors, the research focus of various institutions, and the emergence of new theories from existing institutions. The common tools include VOSviwer, CiteSpace, etc.

CiteSpace is a web-based Java application that is unique and influential in the field of information visualization and analysis. It provides a visual representation of trends in a discipline or field of knowledge, showing how they have evolved over time. This is done by using similarity algorithms to analyze the literature and study the development of various research frontiers. Therefore, the temporal dimension can be employed to clearly outline the evolution of knowledge and the historical scope of literature within a specific cluster. This elucidates the developmental process and trajectory of the field (47).

VOSviewer was released in 2010 by Nees Jan van Eck and Ludo Waltman (48). It is a software tool for creating and exploring maps based on web data. VOSviewer’s advantages are easy to operate and direct aesthetics on visualization (49).

The full documents and citation files from the Web of Science searches are stored in plain text (txt) for analysis. They are then imported into VOSviewer and CiteSpace as a dataset. These tools are utilized for the analysis and creation of visualization maps. In addition, the bibliometrics online analysis website and Prism8 are employed for graphical mapping in this study.

### Search strategy

This study retrieved relevant literature from the Web of Science Core Collection database (WOSCC). WOSCC is widely regarded as one of the world's most authoritative, systematic, and comprehensive databases. This is attributed to its extensive research in biomedical sciences and its superior capability to track older citations compared to Scopus. WOSCC is also recognized for more precise categorization of journals than Scopus (50). The literature search was completed in one day on July 1, 2023 to minimize the impact of frequent updates to the bibliographic database. Science Citation Index Expanded (SCI-expanded) and Social Science Citation Index (SSCI) were used as data sources. We established our search strategy as “TS = [(“cardiovascular disease*” OR “cardiac vascular disease” OR “coronary heart disease” OR “cardiovascular events” OR “coronary artery disease” OR “cardiovascular risk”) AND (“PCSK9”)]”. Literature types included regular and review articles. Time span was between 2006-01-01 and 2023-07-01. The language of publications was restricted to English. The articles were exported and stored in txt format (including full records and cited references) for further analysis (Table 1).

**Table 1:** Summary of data source and selection.

Category	Specific standard requirenments
Research database	Web of Sience core collection
Citation indexes	Sci-expanded, SCIE; SSCI.
Searching period	January 2006–July 2023
Language	English
Searching keywords	[(“Cardiovascular disease*”) AND (“PCSK9”)]
Document types	Articles and review
Sample size	2,747

**Figure 1:**
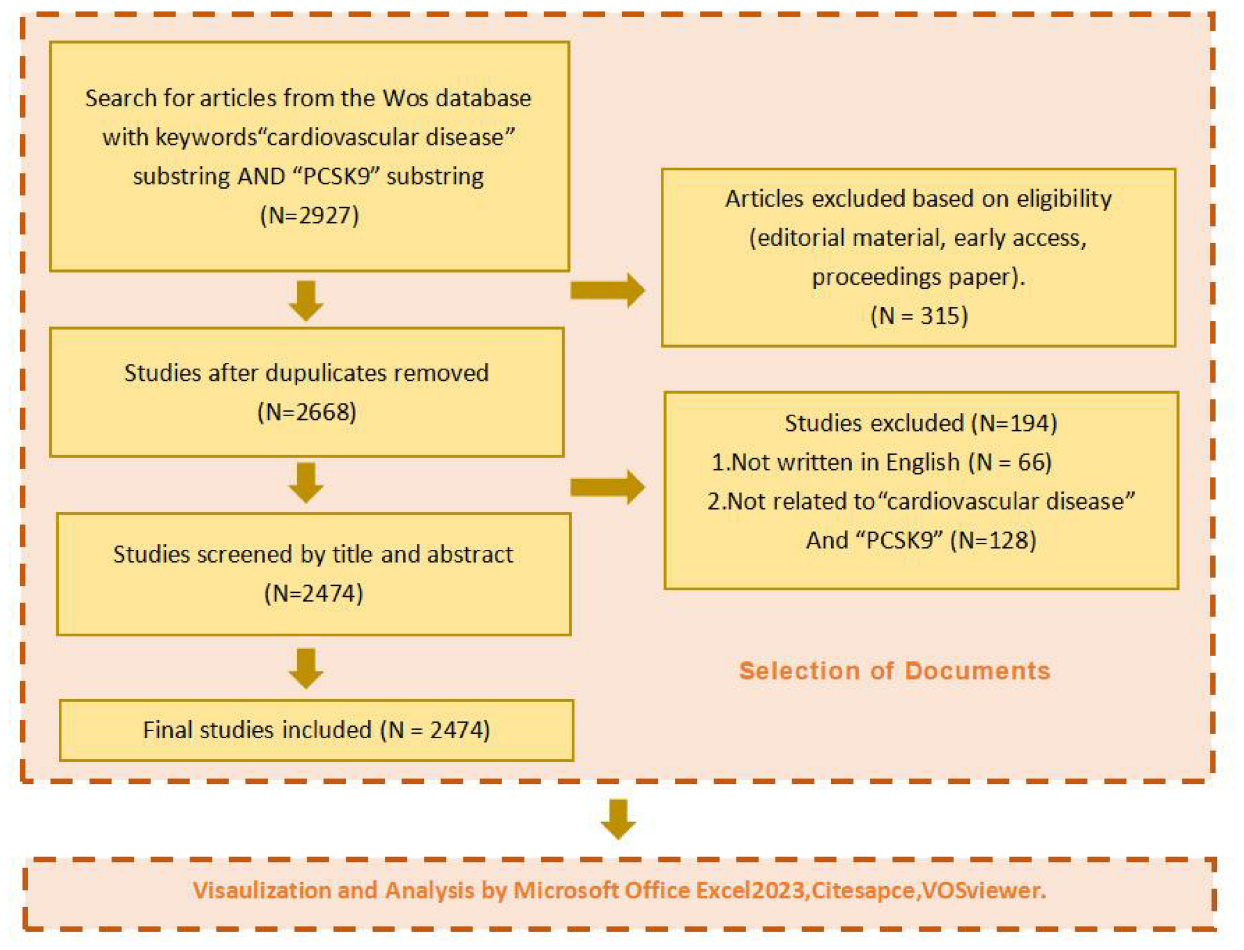
Data cleaning process.

The information on publications was arranged in descending order of their publishing time. To assess the suitability of the studies, they were screened for title and abstract separately. The researchers whose titles and abstracts met the inclusion criteria were included in the full text for searching and evaluation. Figure 1 displays the screening process.

After the screening, we obtained a cluster network containing 2,474 articles. The general information, trends, and focus of new releases in the field of cardiovascular diseases and PCSK9 over the last 18 years were analyzed using bibliometric analysis software (VOSviewer 1.6.19 and CiteSpace 5.7.R5), online bibliometric website (bibliometric.com) and Prism8. Performance analysis and visualization of authors, countries, institutions, journal distributions, most impactful literature, and keywords were conducted.

## Results

### Publication summary

**Figure 2:**
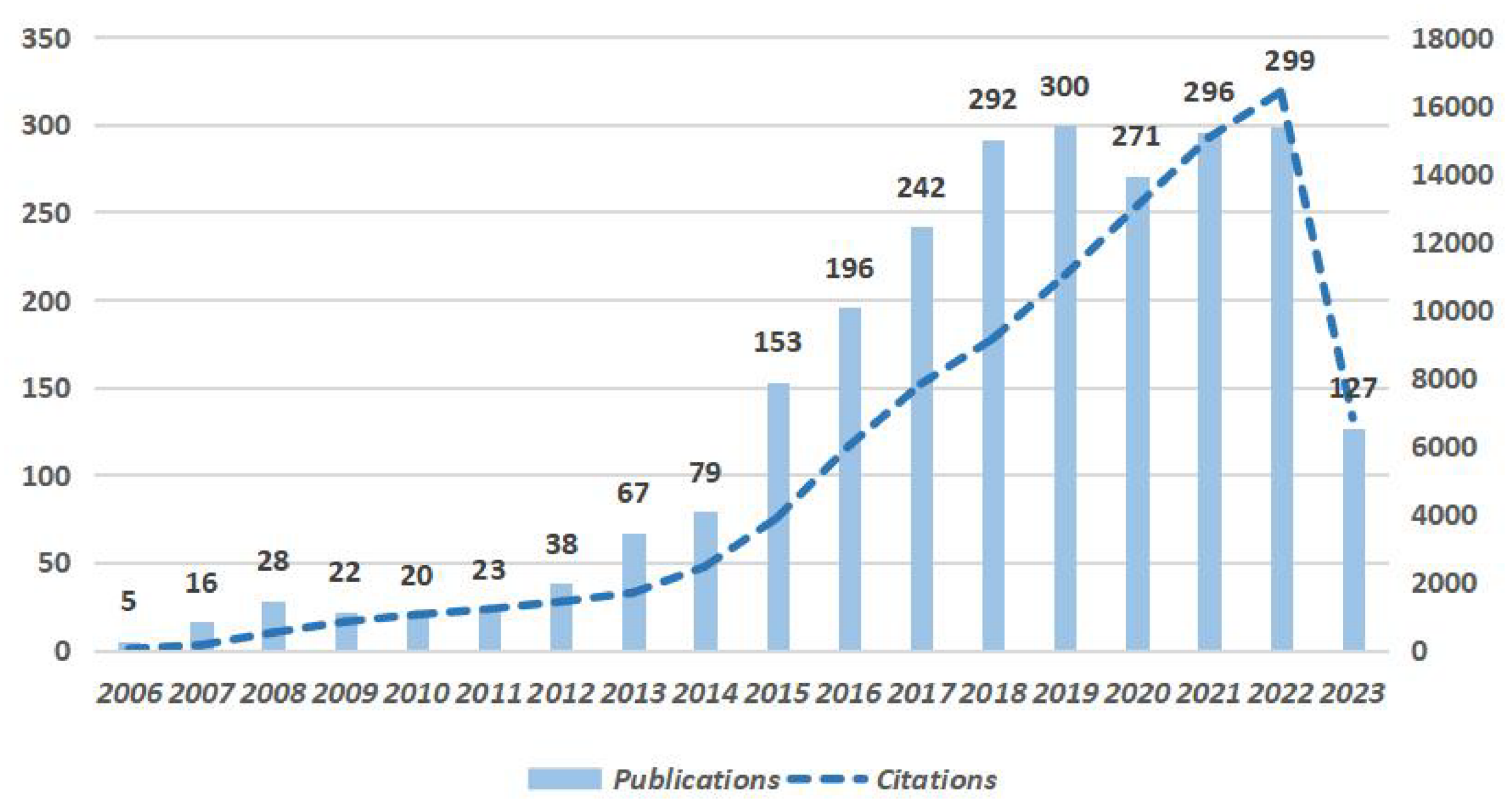
Annual publication and citation trends in global researches in PCSK9 and cardiovascular related fields, 2006–2023.

After de-duplication, a total of 2,474 relevant research papers on PCSK9 and CVD were included, with 1,603 academic papers (accounting for 64.8%) and 871 review (accounting for 35.2%). These researches came from 10,323 authors in 86 countries and 3,243 institutions. These articles were cited a total of 98,330 times. The average number of citations reached 37.73 and were published in 590 journals. 51,003 references from 6,750 journals were cited. Since Cohen, J.C. published the first article in 2006. The number of publications in this field increased from 5 in 2006 to 79 in 2014. The literatures increase year by year. The growth rate of the publications reached 93.7% from 2014–2015 and reached a peak in 2019, with a total of 300 publications this year. More than 250 of researches were published from 2018–2022 (Figure 2). The number of citations increased year by year from 2006–2022 and reached its highest level in 2022. This indicates that the field is maturing and entering a plateau. The detailed numbers of annual papers are presented in Figure 2.

### Analysis of the most productive countries

**Figure 3:**
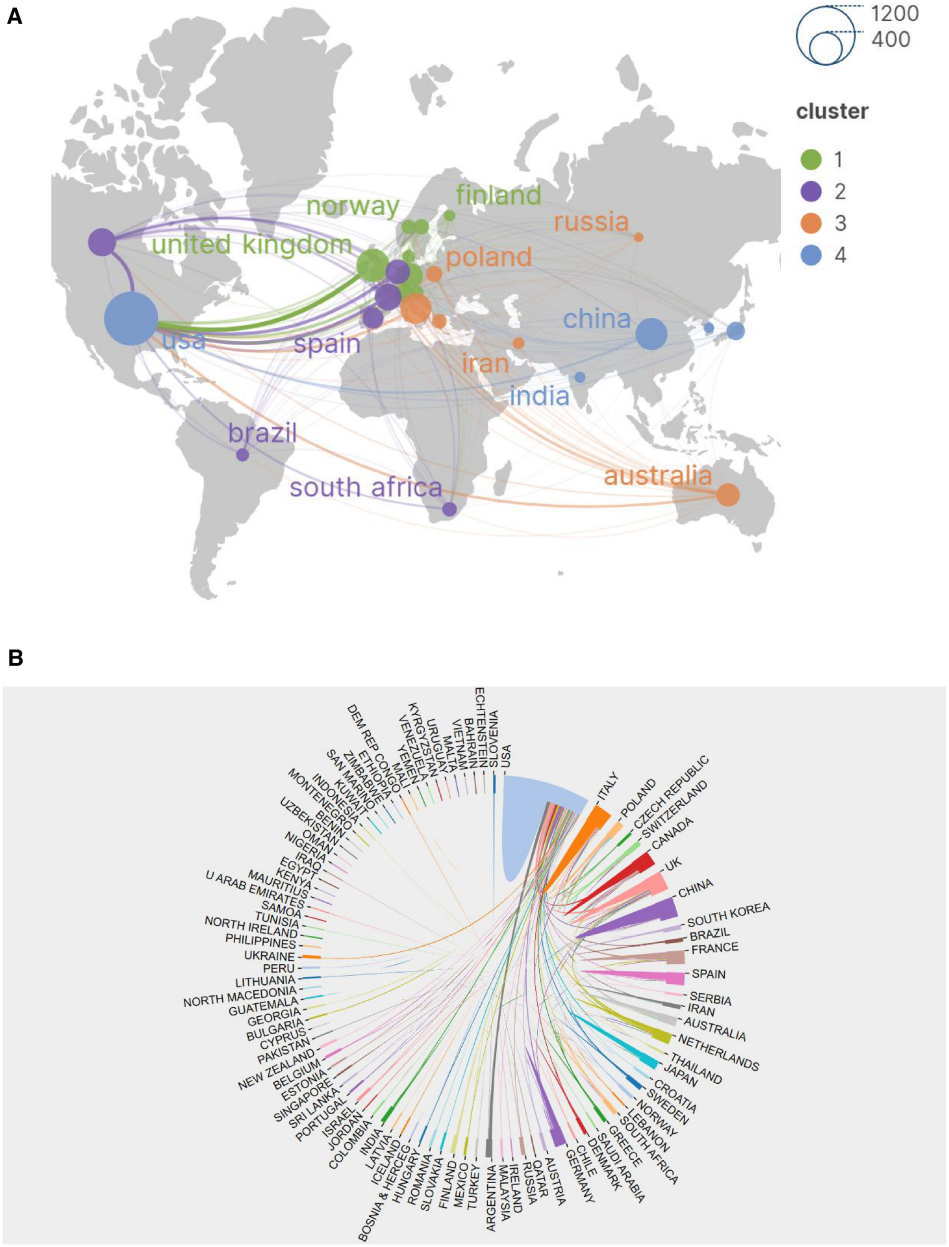
(**A**) Map of intensity of cooperation among countries in the world. (**B**) National cooperation circular chart.

We have mapped network maps using VOSviewer and SCImago Graphica software to understand the top contributing countries and regions and the extensive collaborations among them. The visualized network maps show the numbers of publications and collaborations among countries. The node size indicates the number of published literatures, while the line thickness represents the strength of the links among countries or institutions. The analysis of international cooperation based on 57 countries was displayed in Figures 3A,B. We discover the geographical distribution of publications covering 86 countries/regions on six continents. The circular map depicts the number of publications, with larger sizes indicating a higher number of publications. Links represent the connections among countries or institutions, with thicker links implying more collaborations. Among these countries, 12 have published only one article, while 57 countries have published at least five articles. According to Table 2, the ten most productive countries are the USA (936 papers), England (313), Italy (308), China (302), and Canada (252). The top five countries that work most closely with China are the United Kingdom (link strength of 160), Canada (127), France (121), Netherlands (112), and Germany (88). The United States stands out as the most collaborative country with China, with a link strength of 43 (Figure 3A).

**Table 2:** Top 10 most productive countries.

RANK	Countries	Documents	Citations	Average citation/document
1	Usa	936	57,797	61.75
2	United Kindom	313	27,360	87.41
3	Italy	308	17,597	57.13
4	Peoples R China	302	4,403	14.58
5	Canada	252	21,224	84.22
6	France	227	19,049	83.92
7	Germany	214	14,777	69.05
8	Netherlands	192	18,518	101.17
9	Austrila	175	15,213	96.45
10	Spain	141	8,383	59.45

An analysis of the number of citations for countries shows that the United States has the most citations, followed by the United Kingdom, Canada, France, and the Netherlands. In terms of the average number of citations per article, the Netherlands has the highest with 101.17, followed by Australia with 96.45, England (87.41), Canada (84.22), and France (83.92).

### Analysis of the most productive institutions

**Figure 4:**
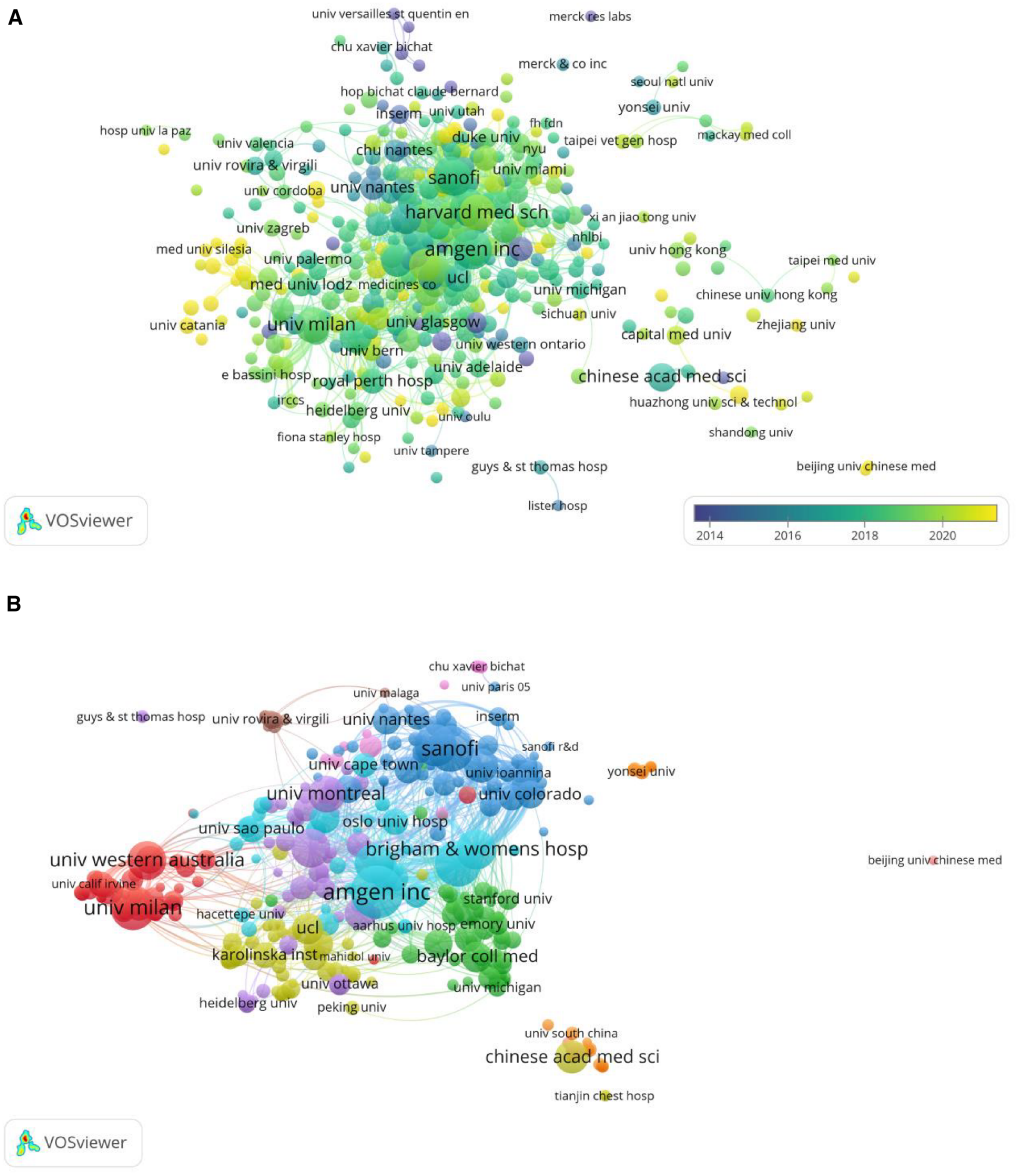
(**A**) Overlay visualization of institution according to the time course. (**B**) Network map of institution collaboration analysis based on VOS viewer.

Through our analysis, we found a total of 3,243 institutions involved in the research on cardiovascular diseases and PCSK9. The top five most prolific institutions were Amgen Inc (114 articles), Harvard Med Sch (96 articles), Sanofi (94 articles), Univ Milan (86 articles), and Imperial Coll London (78 articles). The top ten institutions in terms of contribution and centrality are shown in Table 3. The centrality is usually used to indicate the connectivity of each node. The node with a high mid-centrality indicates that it acts as a key point connecting two or more groups displaying transformation patterns (42). Through CiteSpace software analysis, we found that the top five highest centrality values were Univ Amsterdam (0.17), Capital Med Univ (0.14), Imperial Coll London (0.14), Brigham & Womens Hosp (0.13), and Baylor Coll Med (0.13). To explore when institutional research began and continued, we constructed a timeline graph about institutional research through the visualization feature of VOSviewer. In Figure 4A, the circular nodes size shows the number of publications. The colors of the nodes show the average publications in the first published year in the field. Montreal and Heart Res Inst. started to research earlier, while Mediterranea Cardioctr, Univ Messina, and Chinese academic med sci&peking have conducted more recent research in this field. Figure 4B presents the inter-institutional collaborations. The connecting lines of the nodes represent their collaborations. The intensity of the lines represents the closeness of the collaboration. The institutions that collaborate most closely are Univ Milan and Irccs Multimed, Univ Western Australia and Royal Perth Hosp, both with relative connecting line intensities of 36.

**Table 3:** Top 10 institutions with contributions and centrality.

RANK	Institutions	Documents	Institutions	Centrality
1	Amgen Inc	114	Univ Amsterdam	0.17
2	Harvard Med Sch	96	Capital Med Univ	0.14
3	Sanofi	94	Imperial Coll London	0.14
4	Univ Milan	86	Brigham & Womens Hosp	0.13
5	Imperial Coll London	78	Baylor Coll Med	0.12
6	Brigham & Womens Hosp	73	Sanofi	0.12
7	Regeneron Pharmaceut Inc	73	Univ Penn	0.09
8	Univ Amsterdam	64	Univ Sydney	0.09
9	Univ Western Australia	59	Harvard Univ	0.08
10	Univ Montreal	55	CHU Nantes	0.08

### Analysis of the most productive authors

**Figure 5:**
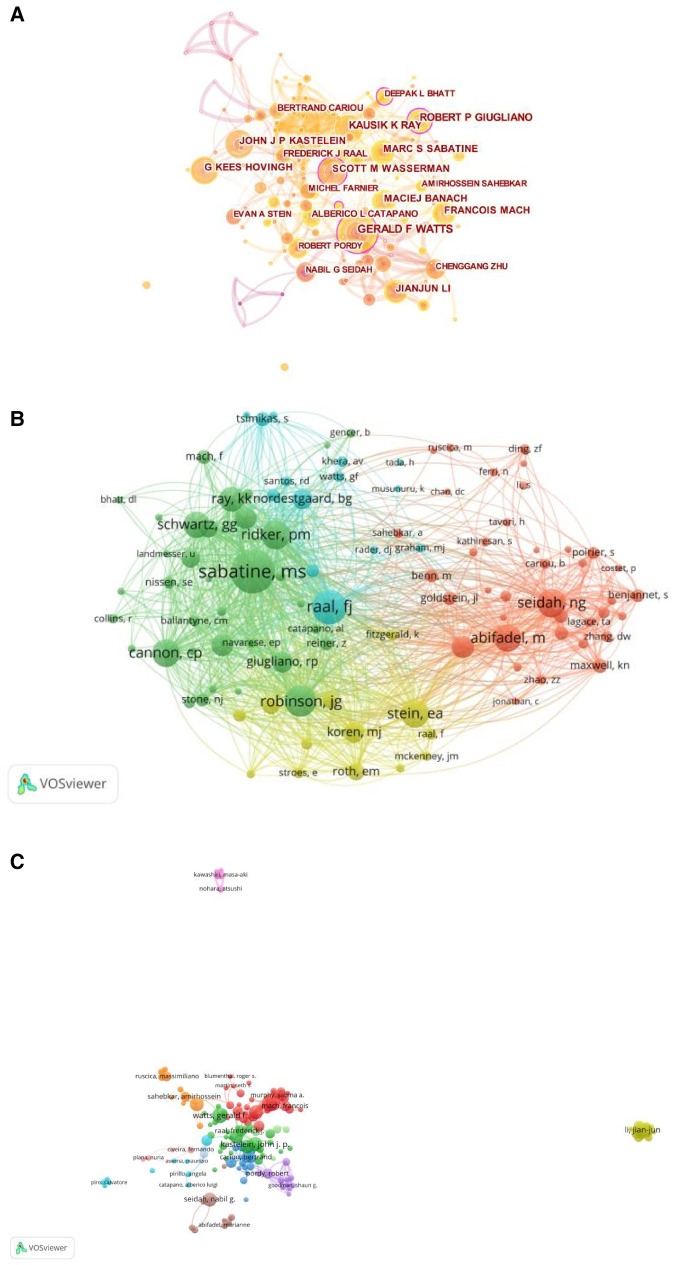
(**A**) Author co-occurrence graph based on Citespace. (**B**) Author co-cited graph based on VOSviewer. (**C**) Author Collaboration Network based on VOSviewer.

We analyzed the results using VOSviewer and CiteSpace to understand the most influential and active authors in the field. The analysis revealed that a total of 10,323 authors contributed to all the 2,474 papers. The number of authors for a single paper, also known as transient indexing, was 8,098, accounting for 78.45% of all authors. Notably, 143 authors have published 10 or more papers in this field. The most prolific author in the field is Gerald F. Watts, with 49 publications and an h-index of 97. Secondly is Marc S. Sabatine with 48 papers (h-index 117) and Robert P. Giugliano with 44 papers (h-index 88) (Figure 5A).

By analyzing Gerald F. Watts' articles, we found that his interests include the diagnosis and treatment of familial hypercholesterolemia, evidence for LDL-induced cardiovascular diseases, and treatment guidelines. He published a total of 49 papers from 2006 to February 2022, receiving a total of 2,006 citations, with an average of 40.94 citations per paper. Gerald F. Watts is affiliated with the Royal Perth Hospital.

Marc S. Sabatine, who ranked second in terms of publication productivity, published 48 papers with a total of 9,376 citations, averaging 195.3 citations per paper. He served at Harvard Medical School and focused on the evolocumab and clinical outcomes in patients with cardiovascular diseases, dapagliflozin, and cardiovascular risk in type 2 diabetes mellitus. There is a close collaboration between Marc S. Sabatine and Gerald F. Watts in the field, particularly in the study of evolocumab's efficacy and safety in reducing blood lipids and cardiovascular events.

**Figure 6:**
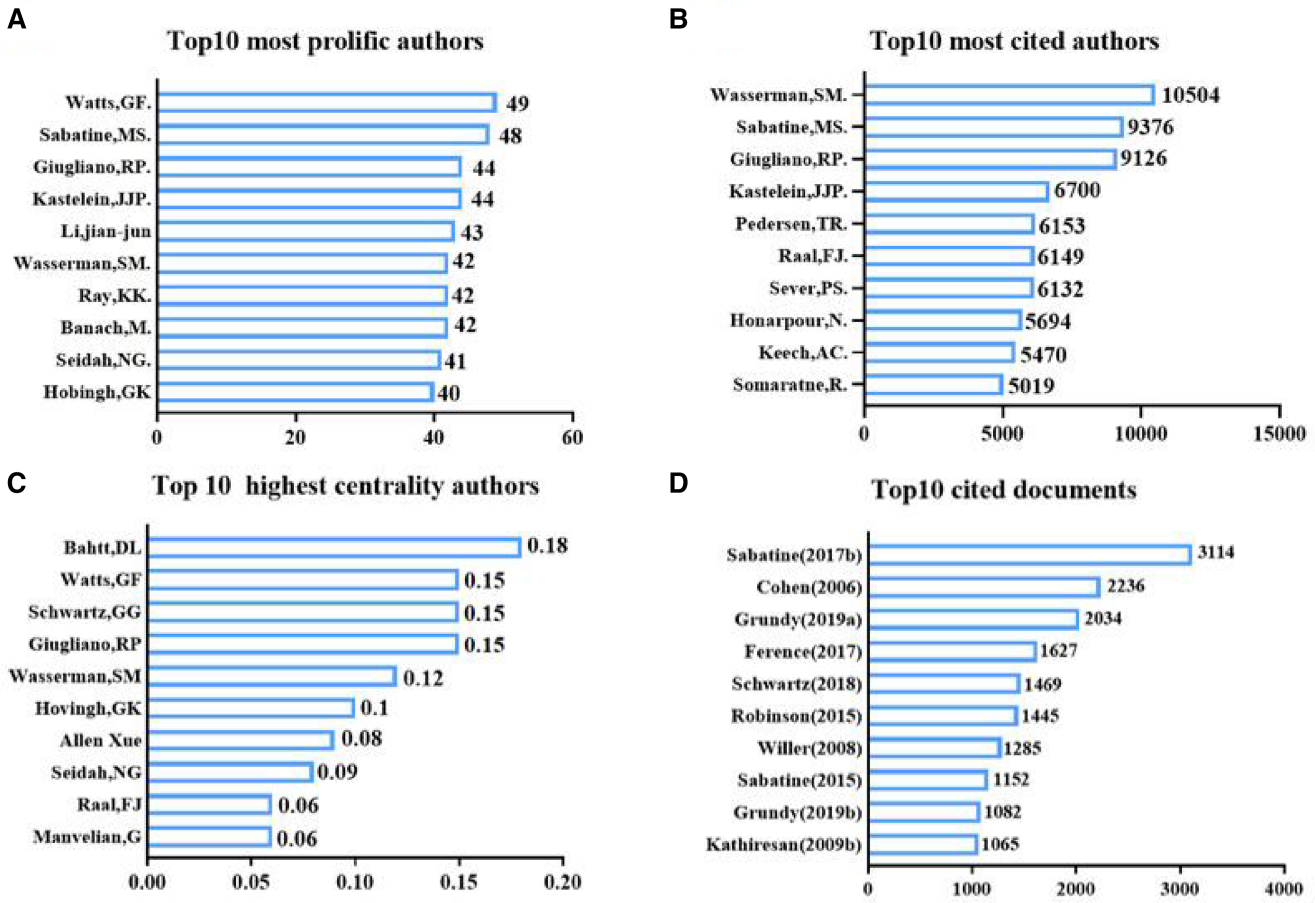
(**A**) Top10 most profic authors (**B**) Top10 most cited author (**C**) Top10 highest centrality authors (**D**) Top10 cited documents.

When analyzing citations, we found a total of 9,325 authors who had one or more citations, accounting for 90.33% of the total 10,323 authors. Among them, 5,965 authors had 10 or more citations (57.78%), and 1,195 authors had 100 or more citations (11.58%). The most influential authors were Wasserman, Scott M. (h-index 55), Sabatine, Marc S. (h-index 117), and Giugliano, Robert P. (h-index 88). Wasserman published a total of 42 papers, receiving 10,504 citations with an average of 250.1 citations per paper. Wasserman's focused on evolocumab (AMG145) for lipid-lowering, a PCSK9 inhibitor, as well as its efficacy and safety for cardiovascular events and *PCSK9* in familial hypercholesterolemia. This author also had a strong collaboration with Marc S. Sabatine in this field. Among the co-cited authors, the most co-cited author is Marc S. Sabatine (h-index 117) with 1,677 citations, followed by F.J. Raal (h-index 58) with 1,236 citations (Figure 6). Additionally, we constructed a network of co-citations (Figure 5B).

In terms of author collaboration, we found that out of the 143 authors who published 10 or more papers. Most of the authors formed only a few collaborative groups. Each group revolved around collaboration with one or two highly prolific authors, as shown in the author's collaboration visualization diagram (Figure 5C). Notably, there were relatively few connections between different groups, suggesting a lack of collaborations between research teams/laboratories in the researches related to cardiovascular diseases and PCSK9.

### Analysis of the most productive source

Two of the most widely used methods to assess the quality of the papers in medical journals are the number of citations and the impact factor (IF). The impact factor (IF) is widely recognized as one of the main indicators that primarily evaluates the quality, importance, and impact of medical journals in their respective disciplines (51, 52). In this research, we found that articles on cardiovascular diseases and PCSK9 were published in 590 journals. Atherosclerosis has the highest publications at 111. We then analyzed the recent impact factors (IF) of the top 10 journals with the highest number of publications. Among these journals, Circulation had the highest IF (Table 4). Additionally, we research the publication volume of these top 10 journals by using Journal Citation Reports (JCR) and found that the majority (60%) were categorized as Q1. In terms of the journal category, the heart and cardiovascular system accounted for 60% of the journal categorization. Finally, when it came to the place of publication among the top 10 journals, among those journals, 7 were in the United States, 2 were in the United Kingdom, and 1 was in the Netherlands.

**Table 4:** Top 10 published journals.

RANK	Sources	Documents	IF
1	Atherosclerosis	111	5.3
2	Journal of clinical lipidology	93	4.4
3	Current atherosclerosis reports	57	5.8
4	Current opinion in lipidology	54	4.4
5	Journal of the american college of cardiology	45	24.0
6	European heart journal	43	39.3
7	Current cardiology reports	41	3.7
8	Journal of the american heart association	35	5.4
9	Frontiers in cardiovascular medicine	34	3.6
10	Circulation	33	37.8

**Figure 7:**
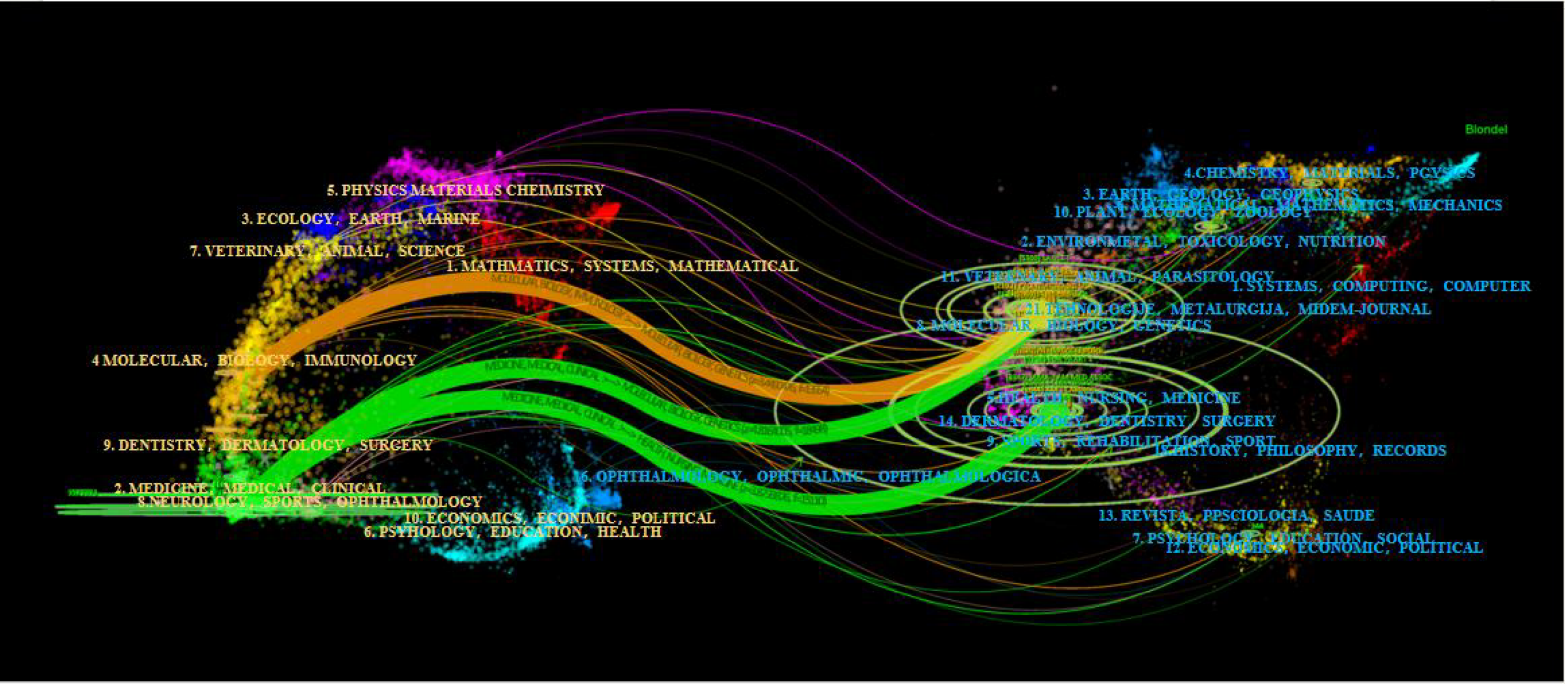
The dual-map overlay of journals on research of PCSK9 and cardiovascular disease researches. The dual-map overlay of journals represents the subject distribution of journals, with the left side of the graph representing citing journals and the right cited journals. The colored lines represent the citation relationship between articles in citing and in cited journals.

The journal dual graph overlay function of CiteSpace was used to construct the disciplinary distribution of academic journals, presented in Figure 7. The area covered by the citing journals is shown on the left-hand side, while the area covered by the cited journals is shown on another side. The dual-map overlay of journals shows 2 major citation paths. The orange path is an independent development pattern, the citing journals mainly belong to MOLECULAR, BIOLOGY, and IMMUNOLOGY, and cited journals mainly belong to TEHNOLOGIJE, METALURGIJA, MIDEM-JOURNAL and MOLECULAR, BIOLOGY, GENETICS. This path indicated that the field has evolved independently from the cited research background topics such as TEHNOLOGIJE, METALURGIJA to the research frontier topics such as MOLECULAR, IMMUNOLOGY. Whereas, the green pathway is a confluent pattern of development, indicating that the two cross-cutting areas evolved into a common research theme, with DERMATOLOGY, DENTISTRY, SURGERY and HEALTH, NURSING, MEDICINE, and the cited themes such as TEHNOLOGIJE, METALURGIJA, MIDEM-JOURNAL, etc. The themes together provide the research basis for the development of the frontiers of NEUROLOGY, SPORTS, OPHTHALMOLOGY, MEDICINE, MEDICAL and CLINICAL. Thus, understanding the development process of the field can provide a good knowledge reference direction for the current research frontiers and grasp the development pulse of the researches.

### Analysis of the top 10 cited documents

**Figure 8:**
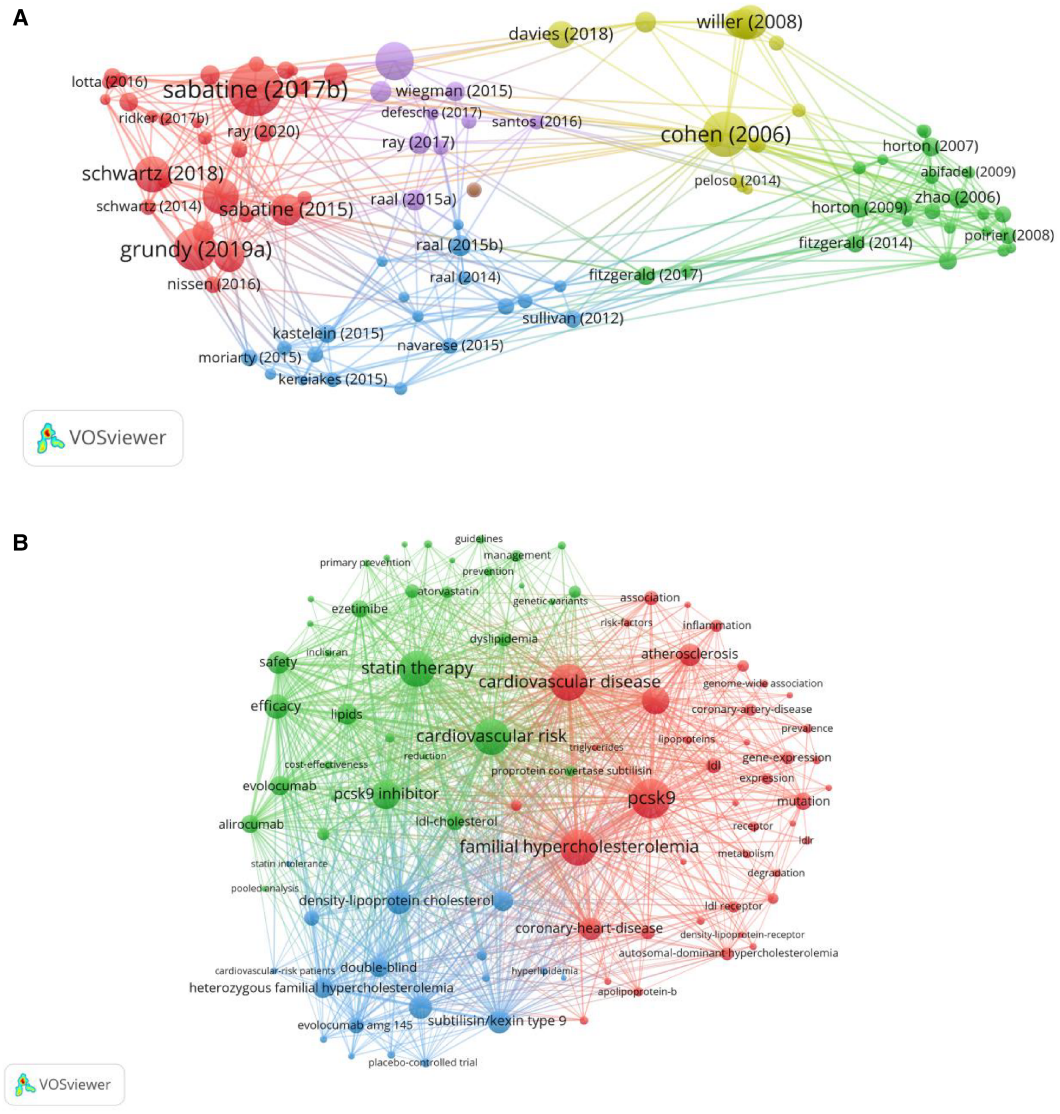

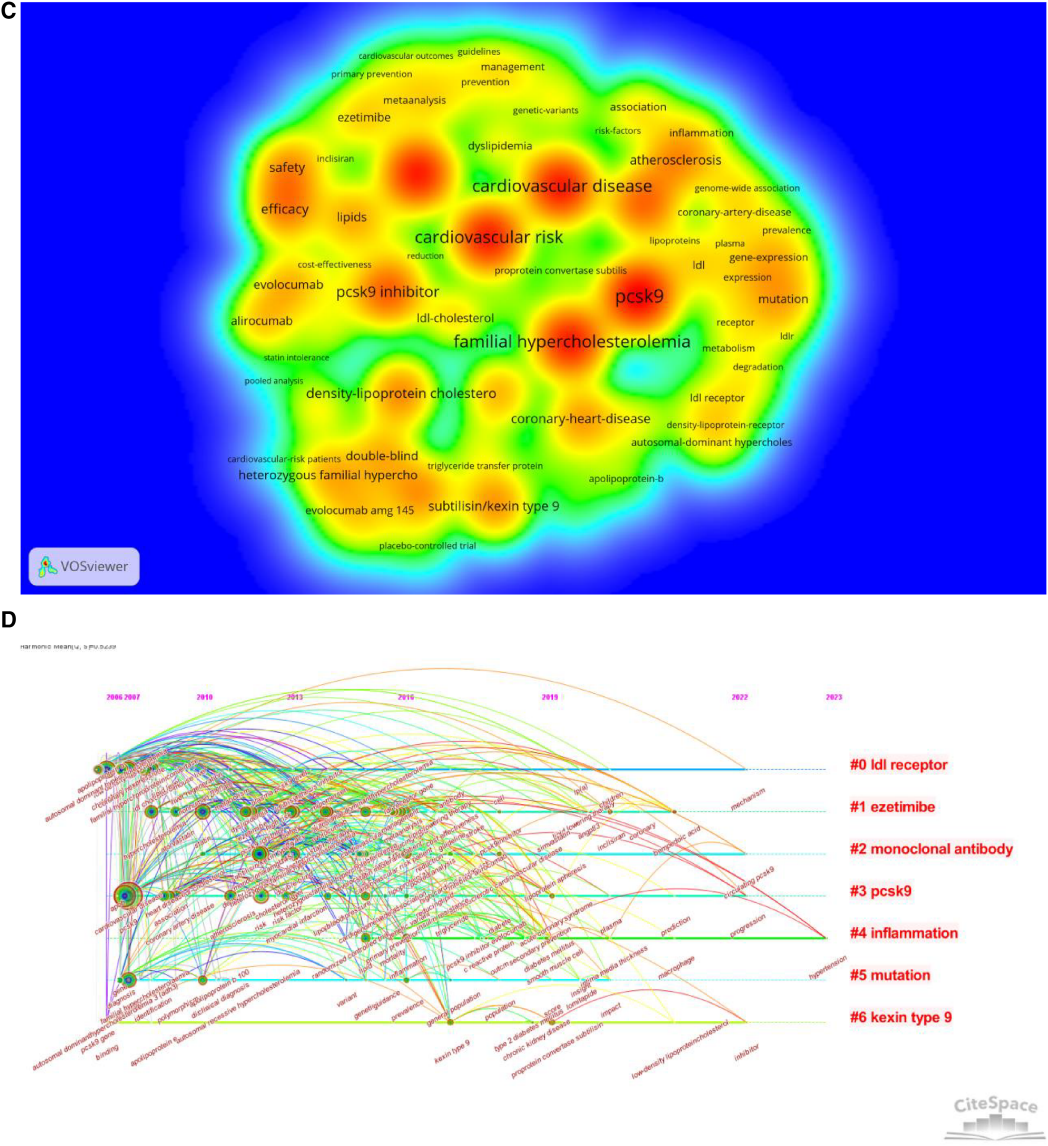

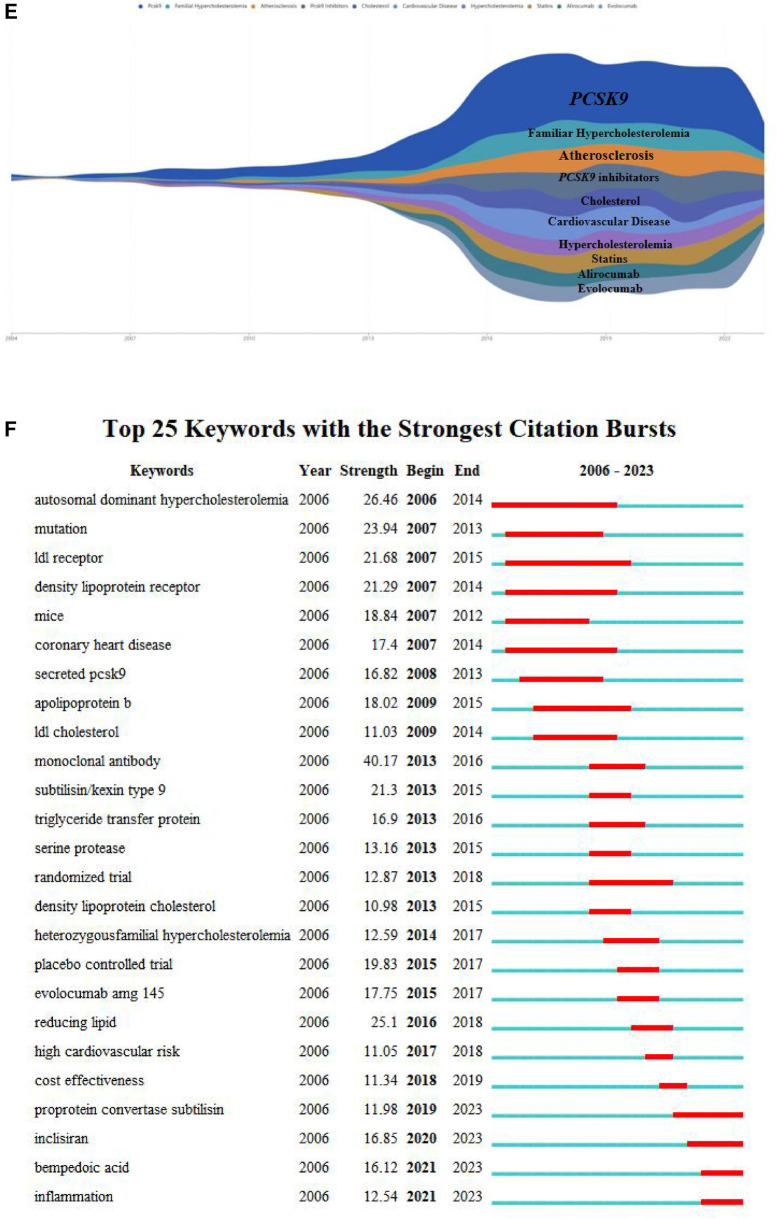
(**A**) Co-occurrence of cited documents based on VOSviewer. (**B**) Keyword co-occurrence and clustering based on VOSviewer. (**C**) Keyword density view based on VOSviewer. (**D**) Keyword timeline based on Citespace. (**E**) PCSK9 technical theme posting trends River Map. (**F**) Keyword burst view based on Citespace.

Citation frequency analysis is a valuable method for evaluating highly cited papers. Citation frequency can reflect the impact of an article in a particular research field (53). In order to explore the most influential research in the field, we analyzed the citation frequency of articles by VOSviewer. A co-occurrence visualization network of article citation counts was derived. The ten most cited articles were published between 2006 and 2019, and all of those were cited more than 1,000 times. Half of these articles were published in the New England Journal, two in Nature Genetics, and three in cardiology journals in the United States and Europe. The first authors of the ten highly cited articles were all from the United States. The cited journals' co-occurrence and top 10 cited documents are charted in Figures 7, 8A.

### Analysis of the keywords

Keywords unite the core and essence of an article. Keywords co-occurrence can identify research hotspots in the field. We analyzed the research hotspots in the literatures by using VOSviewer, analyzing the keyword co-occurrence network and density view in this field. A total of 5,600 keywords, the most frequently occurring keywords were PCSK9 (903 times), cardiovascular disease (804 times), statin therapy (790 times), familial hypercholesterolemia (787 times), PCSK9 inhibitor (539 times), and cholesterol (509 times). The keyword density view is presented in Figure 8C. The more reddish color indicates the higher frequency of keywords.

Clustering keywords can reveal the structure of interconnected research fields. The keyword co-occurrence network mapping analysis was performed on the keyword documents with the top one hundred frequency of occurrence (Figure 8B), in which the nodes that are close to each other and have the same color can be regarded as the same cluster, and three clusters are demonstrated in Figure 8B. Cluster one is in red and contains 42 keywords, including: PCSK9, cardiovascular disease, familial hypercholesterolemia, atherosclerosis, gene expression, mutation, cholesterol, LDLR, etc. Cluster II is in green color and contains 35 keywords, including: PCSK9 inhibitor, evolocumab, alirocumab, statin therapy, cardiovascular risk, safety, efficiency, and so on. Cluster III is in blue and contains 18 keywords, including: statin/kexin type9, double bind, statin tolerance patience, monoclonal antibody, evolocumab amg145, heterozygous familiar hypercholesterolemia, etc.

In order to understand the time point when the keywords appeared and received widespread attention, further analysis was conducted through CiteSpace software and the online bibliometric website. The timeline of the keywords from 2006–2023 is presented in Figure 8D. The more the line color leans towards red means that the keyword has received widespread attention more than recently. While the more it leans towards purple means that the keyword has appeared earlier, and the dotted line means that the keyword has not received much attention in this period. A river diagram about the publication of PCSK9-related topics was presented in Figure 8E, which is based on the time series, counting the number of papers year by year according to different technical subject terms, and forming a trend change diagram within a certain time range. Demonstrating the distribution of PCSK9 in the time dimension can visualize the proportion and change of each related topic in the whole, and also helps to discover potential patterns and associations.

Detecting outbreak words is crucial for identifying evolving research hotspots in an academic field. High citation bursts for articles or keywords suggest that they were widely discussed or utilized during a specific period. To reveal the fastest-growing topics in recent years, we performed keyword outbreak analysis via CiteSpace, Figure 8F presents the 25 keywords with the highest outbreak intensity. The blue line shows the time interval observed from 2006–2023 while the red line shows the duration of the outbreak. The highest outbreak intensity of keyword is monoclonal antibody. Its outbreak intensity is 40.17, which is certified this keyword became a hotspot in 2016 in this field. According to the placebo-controlled trials, autosomal dominant hypercholesterolemia, mutation, LDL receptor, and other keywords are the top 5 highest outbreak intensity and last for many years. Researchers show continuous interest in it. The latest keyword outbreaks include proprotein convertase subtilisin, inflammation, bempedoic acid, and inclisiran, which could be potential frontiers in the field of cardiovascular disease and PCSK9 research.

## Discussion

This bibliometric analysis provides a historical as well as prospective perspective of researches in the field of CVD by helping us clearly sort out the development of PCSK9 related researches. Moreover, it helps researchers to identify the most activity countries, authors, and institutions in the field, also highlights classic works and high-impact journals. This is the first comprehensive bibliometric analysis that reveals the research trends in the related fields of CVD and PCSK9, as far as we know.

### General information for PCSK9 and CVD research

Starting with the first publication in this field in 2006, there was an exploratory start-up process for the following decade. There was a steady increase in document publication number and the extension of document types after 2015. This growth continued after 2018. About 300 articles were published per year. The result shows that the field of research has entered a period of stabilization. The reason for the large increase in articles in 2015 was probably due to the initial introduction of PCSK9 antibodies (23). Monoclonal antibodies targeting PCSK9 were rapidly developed and studied in large clinical programs over the following years. By 2018, three cardiovascular outcome trials demonstrated that PCSK9 inhibitors significantly reduced the risk of major vascular events (13). Even in the context of already being treated with maximal doses of statins, PCSK9 inhibitors lowered plasma LDL-C levels by around 60%, PCSK9 inhibitors, unlike statins, do not result in serious adverse effects, including excessive myalgia, elevated plasma hepatic aminotransferase levels, diabetes mellitus, or neurocognitive adverse events (13). Furthermore, a study (54) showed that evolocumab could be safely and well-tolerated over a median follow-up period of more than seven years. Early intensive use of evolocumab for LDL-C lowering results in a sustained cumulative effect of cardiovascular benefit. To maximize the clinical benefit, it is suggested that early initiation and sustained lowering of LDL-C should be prioritized (54). These trials proved that the PCSK9 inhibitors can reduce LDL-C levels to unprecedentedly low levels (55). It also suggested that more aggressive lipid-lowering strategies should be employed against LDL-C targets.

Inter-country collaboration has played an important role in the field. The United States, as the country with the highest number of articles, occupies a central position in global CVD and PCSK9 research and is at the forefront of this research field in terms of its influence and activity. There is extensive communication and cooperation with several other active countries. USA, Austrila, United Kindom and Canada are the leading countries in terms of publications and collaborative networks, suggesting that these countries may play an important bridging role in research cooperation. In terms of the quality of articles, Netherlands ranked first in terms of citations per article with a high average of 101.17, which indicates that the high quality of articles written by authors from this country is generally valued. On the contrary, China has the fourth highest number of publications but the lowest average of 14.58 citations per article. This highlights the need to prioritize the quality of articles rather than the quantity.

Co-authorship analysis is a useful method for identifying existing partnerships and facilitating the development of potential partnerships (56). Collaboration between researchers helps to explore existing collaborations and find potential collaborators. In the author co-authorship mapping, two highly cited authors, Sabatine, Marc S. and Wasserman, Scott M., have both focused on the field of evolocumab's efficacy and safety in reducing blood lipids and cardiovascular events. They have produced a number of classic works with top ten impacts in the field. The most cited article (Sabatine2017B) was contributed to them with a citation frequency of 3,114 times. This article was a randomized, double-blind, placebo-controlled trial involving 27,564 patients, the median duration of follow-up was 2.2 years. These findings show that patients with athero-sclerotic cardiovascular disease benefit from lowering of LDL cholesterol levels below current targets (55).

In this study, we analyzed the recent impact factor (IF) and journal citation report (JCR) of the top 10 journals with the highest number of publications. Most of the articles were still published in high-impact journals, with 60% classified as Q1. In terms of journal categories, the majority (60%) focused on the heart and cardiovascular systems. Regarding the publishers of the journals, the United States had seven of the top 10 journals, while the United Kingdom had 2 journals and the Netherlands had 1 journal. Notably, there were no high-impact journals in Asia on the list, emphasizing the need to develop influential international journals in the field in Asia.

By analyzing the institutions, we find that the most prolific institution is Amgen Inc., and the institution with the widest reach is Univ Amsterdam. More than half of the top ten activity institutions are universities, indicating that universities are the mainstay of the research field. For the research timeline, Clin Res inst., Montreal and Heart Res inst. are the pioneers in conducting research in this field. Meanwhile, Mediterranea cardiocyte, University Messina, and Chinese Acad Med sci&peking are currently engaged in exploring the latest advancements in this area. Inter-institutional partnerships play a vital role in academia, Univ Milan and Irccs Multimed Univ Western Australia, and Royal Perth Hosp are the institutions that collaborate most closely.

This study checked the top 10 most cited articles and identified their Web of Science categories and research directions, which were mainly in the cardiovascular system and cardiology, lipids, and the use of statins. More than half of these articles revealed that different antibody types of PCSK9 such as Evolocumab and Alirocumab can inhibit PCSK9 and significantly reduce LDL cholesterol levels and reduce the risk of cardiovascular events in the context of statin therapy. These studies also presented that patients with atherosclerotic cardiovascular diseases benefit from reducing LDL cholesterol levels below current target levels, which significantly reduces their risk of developing cardiovascular diseases, and analyzed dyslipidemia and cardiovascular diseases risk from a genetic perspective.

### Research hotspots for keywords

The keywords reflect the core theme and main content of articles, which can provide a reasonable description of the research hotspots. Through the keyword mapping analysis, we find that the prevention and treatment of cardiovascular diseases as well as the diagnosis and treatment of familial hypercholesterolemia are the main research hotspots. Furthermore, recent studies (57) have shown that pretreatment with PCSK9 inhibitor (PCSK9i), at the time of preischemic myocardial ischemia, can play a critical role in prevention of this disease. This is reflected in the reduction of myocardial infarction area and control of arrhythmia during myocardial I/R injury. The mechanism of cardio-protection may be related to the improvement of mitochondrial function and the phosphorylation of conjugated protein 43(Cx43), ultimately leading to improved left ventricle function. Inhibition of PCSK9 reduces the amount of reactive oxygen species (ROS), which result in attenuating mitochondrial damage, reducing mitochondrial swelling, and decreasing depolarization of the membrane potential. These effects provide further protection for the mitochondria. Additionally, the antiarrhythmic effects of PCSK9 inhibitors may be attributed to their interaction with Cx43 (57). However, further investigation is needed to understand the specific pathways involved in these mechanisms.

Researches on familial hypercholesterolemia are also one of the hotspots. *PCSK9* is the third locus associated with familial hypercholesterolemia. Studies demonstrated that mutations in the *PCSK9* caused autosomal dominant hypercholesterolaemia (58). *PCSK9* binds to lipid-associated receptors to regulate metabolic mutations, leading to a higher plasma LDL-C clearance due to a reduction in hepatic LDL-R degradation. In addition, researches show that VLDL-R and ApoER2 are not only the most closely related family members of LDLR but also targets of PCSK9, whose main functions are involved in neuronal development and lipid metabolism (23). This leads to new measures for the diagnosis and prevention of familial hypercholesterolemia from a genetic perspective. Rare gain-of-function mutations in human *PCSK9* cause familial hypercholesterolemia (58). Gene editing *in vivo* is an emerging therapeutic approach that allows DNA modification in patients (e.g., liver). CRISPR base editor is an attractive gene editing modality. The *PCSK9* is a candidate target for *in vivo* gene editing (59). Liver-specific knockdown of this gene using the small interfering RNA (siRNA) inclisiran has been reported to produce a therapeutic effect on patients' lipid levels that lasts for several months (60). In principle, a one-time edit of *PCSK9* might be able to be permanent to bring down blood levels of LDL cholesterol, thereby significantly reducing the risk of cumulative exposure to LDL cholesterol (61). These results may help to discover new therapeutic strategies against familial hypercholesterolemia.

### Timeline viewer and burst analysis for keywords

According to keyword timeline and burst analysis, it is evident that proprotein convertase subtilisin, inflammation, bempedoic acid, and inclisiran may emerge as new potential frontiers in cardiovascular diseases and PCSK9 research. Although the primary action of PCSK9 inhibitors is mediated through the upregulation of LDLR and the subsequent dramatic reduction in circulating LDL-C levels, there is growing evidence that the PCSK9 may have pleiotropic effects. Inflammation has attracted the attention of scholars in recent years. One such role appears to be the modulation of inflammatory mechanisms in atherosclerotic cardiovascular diseases (ASCVD). PCSK9 may play an important role in SRscavenger receptors (SRs) expression, ox-LDL uptake, foam cell formation, and neointimal proliferation. Evidence indicates that PCSK9 is expressed in the infarct border zone. Therefore, targeted therapy against PCSK9, possibly in conjunction with inhibitors of other inflammatory mediators, may provide an effective therapeutic option to reduce the burden of ASCVD (62). This may be able to contribute to the development of new therapeutic strategies for PCSK9 application in the cardiovascular field as well as in other areas.

In summary, our study explores the journey of the global CVD and PCSK9 related fields from the emergence of research to the present. By analyzing these articles, we describe the major advancements in PCSK9 research regarding topics, institutions, countries, authors, and study types in cardiovascular disease research, offering researchers a quick understanding of the major research directions in this field. Our study also highlights the potential hotspots and frontiers of research.

### Limitations

In this study, the language of all articles included was limited to English. We may miss some high-impact articles related to CVD and PCSK9 but published in other languages. Second, we only searched the web of scientific databases. The type of publication only included regular and review articles. There were also limitations in the scope of literature source access. Thirdly, there may be contradictions in various aspects, such as an institution using different names at different times. Bias may result from several factors, including disproportionate or inappropriate citations due to institutional bias, linguistic bias, self-citation, bias of the powerful, internal bias, and intentional omission of citations for reasons such as competition (63, 64).

## Conclusion

The importance of PCSK9 application in CVD is increasing, as indicated by the significant increase in the number of annual publications globally. Among all countries, the United States has the largest number of publications and the greatest impact. This study identifies the major researchers and institutions involved in PCSK9 and CVD research worldwide. Atherosclerosis is the most prolific journal in this field, while Circulation has the highest impact factor. Hot topics include prevention of cardiovascular diseases, lipid-lowering therapy, diagnosis, and prevention of familial hypercholesterolemia. Future researches may focus on inflammation, bempedoic acid and inclisiran. These results offer a comprehensive view for new researchers and policymakers in this field. In conclusion, the application of PCSK9 in the field of CVD is of growing importance, and these insights provide valuable perspectives for future studies.

## Data Availability

The original contributions presented in the study are included in the article/supplementary material, further inquiries can be directed to the corresponding author.
